# Supplementary material for: Berberine Depresses Inflammation and Adjusts Smooth Muscle to Ameliorate Ulcerative Colitis of Cats by Regulating Gut Microbiota
Source: Microbiol Spectr. 2022 Oct 26;10(6):e03207-22. doi: 10.1128/spectrum.03207-22 (PMC9769923; doi:10.1128/spectrum.03207-22)
Supplement: Supplemental file 1 — Fig. S1 and S2. Download spectrum.03207-22-s0001.pdf, PDF file, 0.2 MB [file spectrum.03207-22-s0001.pdf]

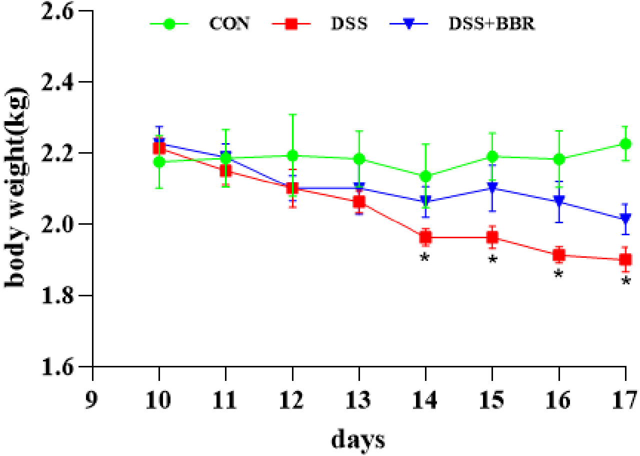

SUPPLEMENTARY FIG 1 The body weight of the cats.(vs. CON*P < 0.05).

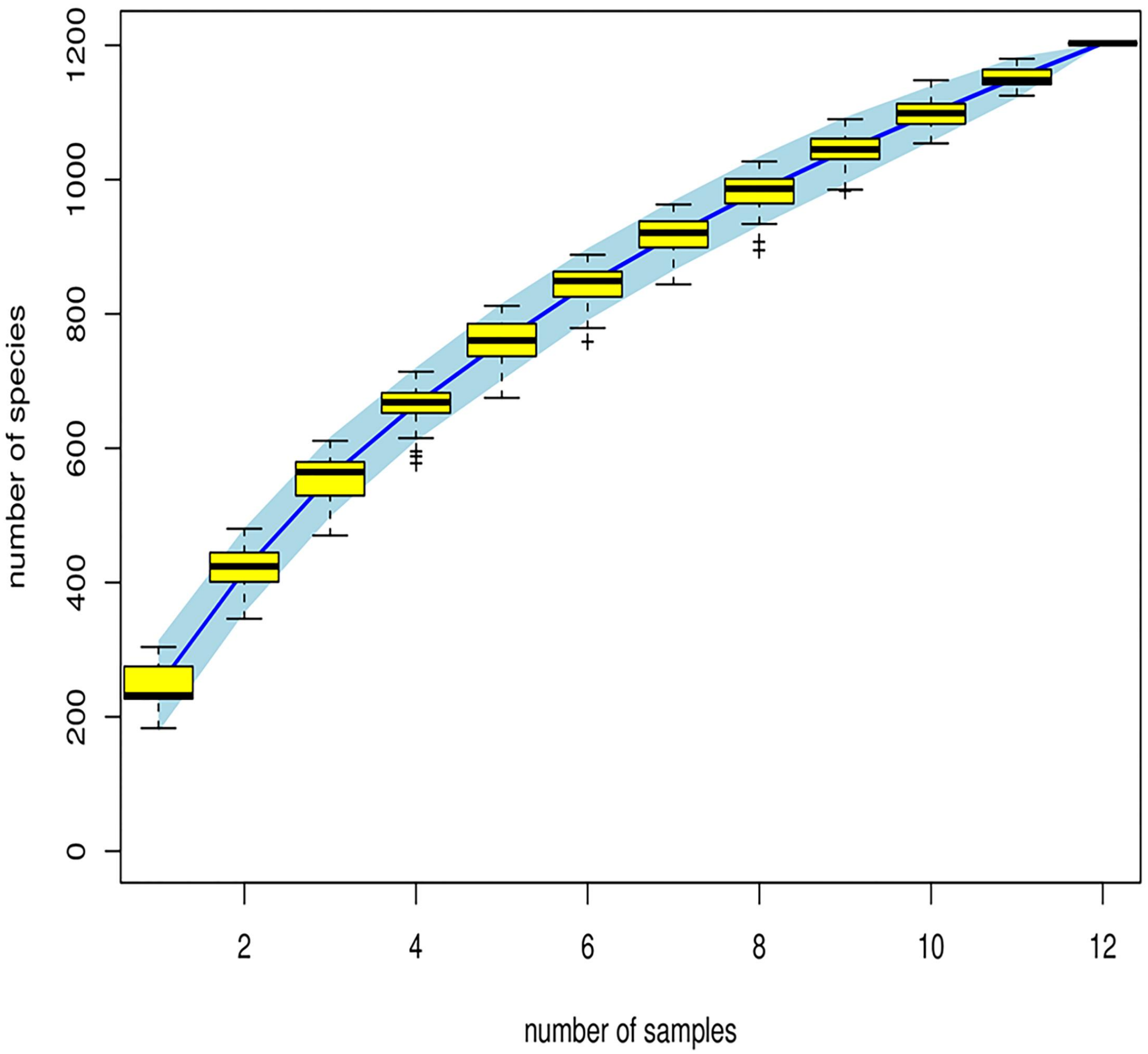

SUPPLEMENTARY FIG 2 Accumulation curves. The curves reflect the rate of increase in the number of species observed in the sample as the sample size increases, and the blue shading reflects the confidence interval of the curve.
